# Effects of Vibration-Based Generation of Timing of Tactile Perception on Upper Limb Function After Stroke: A Case Study

**DOI:** 10.7759/cureus.50855

**Published:** 2023-12-20

**Authors:** Ayaka Awaji, Takeshi Fuchigami, Rento Ogata, Shu Morioka

**Affiliations:** 1 Department of Physical Therapy, Faculty of Health Sciences, Kio University, Nara, JPN; 2 Neurorehabilitation Research Center, Kio University, Nara, JPN; 3 Department of Rehabilitation, Kishiwada Rehabilitation Hospital, Osaka, JPN; 4 Stroke Rehabilitation Research Laboratory, Kishiwada Rehabilitation Hospital, Osaka, JPN; 5 Department of Neurorehabilitation, Graduate School of Health Sciences, Kio University, Nara, JPN

**Keywords:** a case study, rehabilitation, sensorimotor deficits, stroke, upper limb, tactile perception

## Abstract

Sensorimotor dysfunction of the fingers and hands hinders the recovery of motor function post-stroke. Generally, hemiplegic patients are unable to properly control the dynamic friction generated between their fingers and objects during hand/finger muscle activity. In addition to sensory information, a sense of agency generated by the temporal synchronization of sensory prediction and sensory feedback is required to control this dynamic friction. In the present study, we utilized a novel rehabilitation device that transmits real-time fingertip contact information to a transducer in a case of stroke hemiplegia with sensorimotor deficits and stagnated hand/finger motor performance. Post-intervention, the patient's upper extremity motor function score (FMA-UE), which had previously been in a state of arrested recovery, improved from 51/66 to 61/66, especially in the wrist joints. Excessive grip force during object grasping and frequency of falling objects was notably decreased post-intervention. We believe that rehabilitation tasks using perceptual generation via transducer will be a new tool for the rehabilitation of post-stroke hand/finger sensorimotor deficits.

## Introduction

Only 5%-20% of patients fully recover from stroke-related upper limb motor paralysis [1,2], and 25%-74% require assistance with activities of daily living [3]. Of these, functional recovery is the slowest in the distal parts of the body [4-6]. Even after partial recovery from upper limb paralysis, muscle activity patterns during distal joint movements often remain abnormal [7]. Moreover, unusual movement patterns occur in the distal parts of the body, in conjunction with the proximal parts, when performing reaching movements of the upper extremities [8]. Particularly, paralysis of the fingers and hands severely limits activities of daily living, resulting in a decreased quality of life [9,10]. This is because fine hand motor skills are necessary for manipulating tools (e.g., using a fork, buttoning a shirt, or turning a doorknob) in activities of daily living [11].

A common symptom of hemiplegia is sensory deficit of the hand, which is closely related to upper extremity motor function [11]. The sensory function of the hand is responsible for detecting small fluctuations and errors in muscle contraction and regulating movement [12,13]. When somatosensory feedback in the afferent tract is interrupted or attenuated while one is grasping an object, the central nervous system sends signals that cause excessive gripping forces, thereby increasing force fluctuations during movement and causing the object to fall [14]. This is referred to as hand/finger sensorimotor dysfunction, which is considered a barrier to recovery of motor function.

Visual [15-17], electrical [18,19], and auditory [20] stimulation have been used as rehabilitation methods for sensorimotor dysfunction in hemiplegic patients. Although visual feedback is effective in capturing targets, it undeniably lacks somatosensory information that allows real-time information tracking. This results in inefficient motor control due to motion uncertainty, such as increasing motor output to avoid accidentally dropping hand-held objects [21-23]. In hemiplegic patients, it is difficult to properly control the friction between the fingers and the object (dynamic friction) with hand/finger muscle activity. Even with electrical or auditory stimuli, it is difficult to detect continuous feedback corresponding to dynamic friction. Smooth control in response to feedback stimuli, which is required to move precisely according to the shape of the object, also remains difficult despite these stimuli. Furthermore, dynamic friction requires perceptual information of the hand muscle activity, and generation of a sense of agency, which is essential for smooth motor control, requires temporal synchronization of information between motor intention and sensory feedback [24].

When a person touches an object, receptors on the hand and fingers perceive it as a vibration from the frictional information of the object. Tanaka et al. developed a wearable device that detects such vibrations and provides real-time feedback of the minute changes in friction generated at the fingertips [25]. These vibrations have been shown to contribute to roughness perception in another study [26]. Higashi et al. also found that vibration affects the perception of hardness [27]. Based on these findings, a close relationship between vibration and tactile perception can be suggested. Rehabilitation medicine for post-stroke hemiplegia requires compensation for the constant changes in tactile information between the object and fingers. In this way, it may be possible to reorganize the fine motor movements of patients with hand/finger sensorimotor dysfunction.

In the present study, we utilized a novel rehabilitation method for a patient with post-stroke sensorimotor dysfunction, which resulted in stagnated hand/finger motor ability. The device used recognizes contact information from the muscle bellies of the fingers via transducer and transmits this information in real time. We describe the procedure and the results of the efficacy evaluation in this paper.

## Case presentation

**Figure 1:**
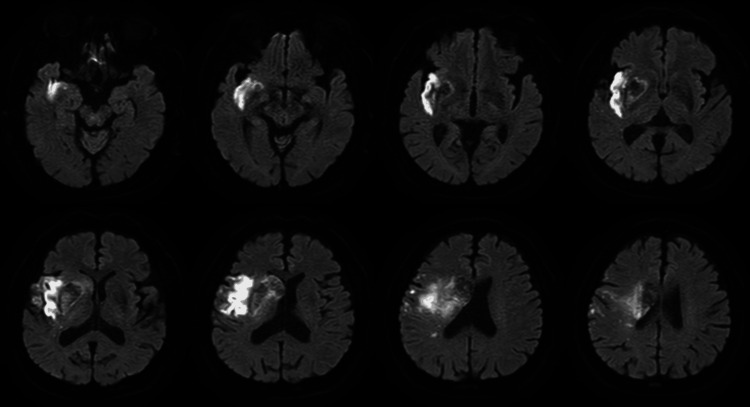
MRI at the time of onset showing hemorrhagic cerebral infarction in the region of the right middle cerebral artery.

The patient was a 70-year-old male diagnosed with paralysis of the left upper and lower extremities due to a hemorrhagic stroke (Figure 1). Following acute treatment, he was transferred to our hospital for rehabilitation on the 20th day post-ictus. On initial evaluation, the 9-Hole Peg Test (9-HPT), a hand motor performance test, was completed in 32.6 seconds using his right hand; however, the test could not be performed using his left hand with noted difficulty with manipulating objects. The Fugl-Meyer Assessment Upper Extremity (FMA-UE) total score was 51/66, with 0/10 function of the left wrist joint, indicating significant impairment. Based on the Box and Block Test (BBT), which assesses upper extremity endurance and fine motor skills, 33 blocks were moved with the right hand, and 17 blocks were moved with the left hand. The Action Research Arm Test (ARAT), which assesses object manipulation, was 44/57. Grip strength was 14.8 kg for the right hand and 6.7 kg for the left hand. In addition, the left upper extremity was not involved in eating activities. The Stroke Impairment Assessment Set (SIAS) score, which was used for sensory testing, was 4/12. Notably, superficial sensation was moderately reduced, whereas deep sensation was severely reduced.

The rehabilitation program during hospitalization included physical therapy and occupational therapy, each provided for one hour per day (seven times per week). After the initial evaluation, the patient underwent ankle range of motion exercises, standing and gait training, large peg manipulation, therapist grasping, and small pea pinching for one month. As a result, 9-HPT improved to 31.3 seconds for the right hand and 86.6 seconds for the left hand, BBT improved to 29 blocks for the right hand and 20 blocks for the left hand, ARAT improved to 50/57 points, and grip strength improved to 16.3 kg for the right hand and 8 kg for the left hand. However, FMA-UE showed no improvement, with a score of 51/66 points. Regarding sensory function, SIAS showed a total score of 8/12 (two each for light touch sensation in the upper extremity, light touch sensation in the lower extremity, position sensation in the upper extremity, and position sensation in the lower extremity). Although passive sensory function showed improvement, the patient exhibited hand/finger sensorimotor dysfunction, resulting in excessive force during object grasping, significant fluctuations during object manipulation, and accidentally dropping objects. This stage was defined as pre-intervention (X). At this time, the Mini-Mental State Examination score was 25/30 points; the Trail Making Test results were (a) 79.0 seconds, (b) 217.0 seconds; and the Behavioral Inattention Test score was 139/146 points, indicating no cognitive dysfunction that would make intervention difficult.

Intervention protocol

For sensorimotor dysfunction of the left hand, the patient performed motor tasks while wearing a device (Yubi-Recorder, Tech Gihan Co., Ltd., Kyoto, Japan) that detects contact information on the finger muscle bellies using a vibrator and transmits real-time information five times a day for 15 days (these 15 days were immediately after X, defined as X+15). The wearable device can detect any vibration of the skin that occurs when an object is touched, thereby measuring vibration information. Specifically, it can detect uneven, flat, curved, and random surfaces; capture tactile stimuli from objects of any shape; and can adequately handle multidirectional motion. By attaching a vibration sensor to the distal interphalangeal (DIP) joint of the index finger and modulating the sensor information to a frequency perceived by humans, vibration information can be presented using an oscillator. In the present case, the sensor was attached to the DIP joint of the index finger of the affected limb, allowing detection of the tactile information of the skin as vibration. Moreover, by attaching an oscillator to the left clavicle, the vibration stimulus was presented in timed synchronization.

When the patient’s hand touches an object, the vibrator in the collarbone vibrates in real-time, with varying intensities depending on the feel of the object. We instructed the patient to feel the different vibrations and associate these differences with the objects they feel at their fingers.

**Figure 2:**
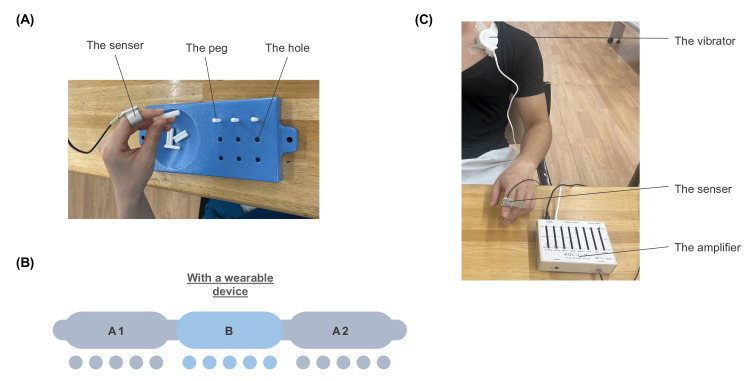
Intervention procedures. (A) The intervention task was to insert a peg into a pegboard and was performed with the left hand, the affected limb (the 9-Hole Peg Test). The task was performed five times daily for 15 days. (B) Experimental protocol. The ABA design was used for the intervention design: the intervention task without a wearable device (Phase A1), the intervention task with a wearable device (Phase B), and the intervention task without a wearable device (Phase A2). Each period lasted five days. (C) The wearable device. A tactile sensor was attached to the distal interphalangeal joint of the index finger of the affected limb. The sensor detected vibration information on the ventral skin of the index finger, which was presented synchronously from a vibrator attached to the left clavicle.

**Figure 3:**
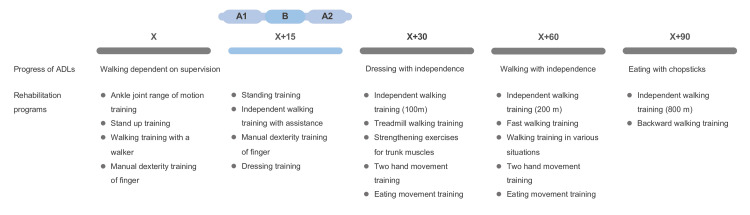
Progress of hospitalization. The rehabilitation program other than intervention and the progression of activities of daily living (ADLs) from admission to discharge are shown. The time from admission to just before intervention was X, during intervention was X+15, 30 days after X was X+30, 60 days after X was X+60, and 90 days after X was X+90. Over time, the patients moved from manual dexterity training with the left hand to two-hand movement training, and from simple walking training to applied walking training. At X+90, the patient was able to eat using chopsticks and was discharged home. An ABA design, with each period lasting for five days, was used for the intervention design at X+15: intervention task without the wearable device (A1), intervention task with the wearable device (B), and a second intervention task without the wearable device (A2).

For the intervention task, the 9-HPT (Sakai Medical Corporation, Tokyo, Japan) was utilized, wherein the patient was instructed to insert a peg into a pegboard using the left hand (Figures 2A-2C). An ABA design, with each period lasting for 5 days, was used for the intervention design: an intervention task without the wearable device (A1), an intervention task with the wearable device (B), and a second intervention task without the wearable device (A2). In addition to this task, standing, walking, and dressing training were performed (X+15). The patient was then discharged 90 days after pre-intervention (X+90). The rehabilitation care provided is shown in Figure 3.

Prior to the intervention, we confirmed that the patient had the motor function to perform the task. We also confirmed that he had no cognitive dysfunction, communication disorder, sudden seizures such as epilepsy, and poor general condition. The study was conducted in accordance with the tenets of the Declaration of Helsinki and was approved by the Ethical Committee of Kishiwada Rehabilitation Hospital (approval number 2023-06), where the patient was admitted. The patient was fully informed verbally and in writing of the purpose, content, and procedures of the study, and his informed consent was obtained.

Evaluation and analysis

During the Intervention

The number of errors was noted during the intervention task using the 9-HPT. An error was defined as failure to grip the peg and the peg falling off the table. If an error occurred, the assessment would be restarted from the beginning. The time from when the first peg was grasped to when all pegs were removed and placed on the plate (Figure 2A) was measured using a stopwatch (Doritec Corporation, Tokyo). The measurements were done by an occupational therapist blinded to the design of this study, and the average of the tasks performed five times was calculated for each day.

Averages were calculated for each five-day period for periods A1, B, and A2, and the slope (a) for each period was calculated using the linear function equation (y=ax+b). Additionally, cross-lag correlation analysis was used to analyze the temporal relationship between the number of errors and the time required to complete the task. The procedure used by Canerio et al. was similarly used for the analysis of the present study [28]. All data points were analyzed with lags -5 to 0 and 0 to +5 using the open-source software (https://clinicalresearcher.org/) developed by Borckardt [29]. The processing of the analysis was also performed following a case-based time series [29]. It should be noted that lag -5 indicates the correlation between day 1 of period B and day 1 of period A, and lag +5 indicates the correlation between day 5 of period B and day 5 of period A2. The data for the two variables were analyzed using non-parametric methods because normal distributions could not be confirmed.

Comparison Before and After X+15

FMA-UE, BBT, ARAT, SIAS, and grip strength measurements were performed pre- and post-intervention (X+15). The Motor Activity Log (MAL), a questionnaire consisting of Amount of Use (AOU) and Quality of Movement (QOM) items scored on a 6-point scale (0 = not used, 5 = normal), was also used to assess subjective use of the affected hand during hospitalization.

In addition, the patient's sense of agency when moving the affected hand was assessed. During the pin manipulation task, he was asked to report his reflection, and his sense of agency was quantitively measured using psychophysical methods after performing the task. A personal computer (FUJITSU LIFEBOOK UH90/C3, Tokyo, Japan) with a 13.3-inch display was used for all tasks and data recording. In previous studies, patients were instructed to freely manipulate the dots (40 pixels) on the screen with the affected limb within four seconds. The dot movements were intermixed with pre-recorded "other" movements and were randomly inserted every 10% of their manipulation 0-100% of the time. The number of trials was 110 in total, with four seconds of manipulation per trial (10 trials of each 10% of 0%-100%). These tasks were created using MATLAB and PsychToolbox (MathWorks, Natick, MA, USA). With each trial, patients were asked if they felt in control and if they felt uncomfortable with the movement of the dots [30,31]. This was similarly done for our patient in the present study. The action-outcome regularity detection probability for each control condition was calculated. Logistic curves were fitted to the action-outcome regularity detection probability using the following formula [32,33]:

$P(t)=\frac{1}{1+exp(-\alpha (t-t_{PSE}))}$

where *t* is the control level, i.e., the ratio of the participant’s finger movements to the target dot movements; *P(t)* is the action-outcome regularity detection probability; α indicates the steepness of the fitted curve; and *t*_PSE_ indicates the control level under which the probability of detecting action-outcome regularity is 50%. The analyses were carried out with MATLAB R2016a (MathWorks, Natick, MA, USA) using the generalized linear model function in the Statistics and Machine Learning Toolbox.

The slope of the logistic curve was used as an indicator of a sense of agency [30,31]. The slope indicates the strength of the sense of agency. If the slope is steep, the agency of the patient’s movement is more likely, while if it is gradual, the agency of the patient’s movement becomes less likely.

Results

**Figure 4:**
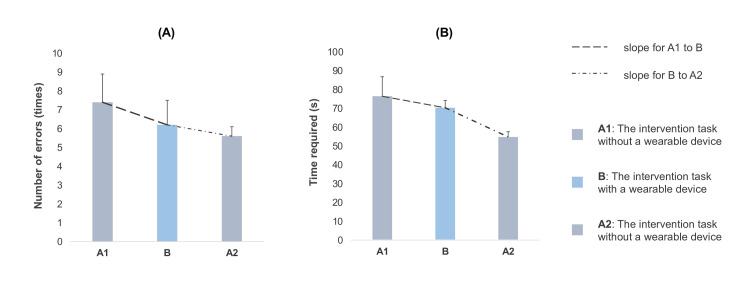
Result of the 9-Hole Peg Test. Averages were calculated for each 5-day period for periods A1, B, and A2, and the slope (a) for each period was calculated using the linear function equation (y=ax+b). The bar is standard deviation. The mean number of errors (times) was 7.4±1.5 (means ± standard deviation) in period A1, 6.2±1.3 in period B, and 5.6±0.5 in period A2 (A). The mean time required (seconds) was 76.4 ± 10.4 in period A1, 70.3 ± 3.9 in period B, and 54.8 ± 2.8 in period A2 (B). Both the mean and standard deviation of the number of errors (A) and time required (B) decreased over time. For the number of errors (A), the slope was -1.2 from period A1 to B and -0.6 from period B to A2. For the time required (B), the slope was -6.1 from period A1 to B and -15.6 from period B to A2. All values were negative, indicating a decrease in the number of errors and a reduction in the time required. The slope of the number of errors (A) was higher than that of the time required from period A1 to B, whereas the slope of the time required (B) was higher than that of the number of errors from period B to A2.

The patient reported no discomfort or significant adverse events due to the intervention. The 9-HPT results (Figure 4) showed that the mean number of errors (times) was 7.4 ± 1.5 in period A1, 6.2±1.3 in period B, and 5.6±0.5 in period A2. The mean time required (seconds) was 76.4 ± 10.4 in period A1, 70.3 ± 3.9 in period B, and 54.8 ± 2.8 in period A2 (means ± standard deviation). For both errors and time required, the mean and standard deviation decreased over time. The slope (a) for the number of errors was -1.2 from period A1 to B and -0.6 from period B to A2, and the slope for the time required was -6.1 from period A1 to B and -15.6 from period B to A2. In comparison, the slope of the number of defects was greater than that of the time required from period A1 to B, whereas the slope of the time required was greater than that of the number of defects from period B to A2. Cross-lagged correlation analysis showed that the correlation between the number of errors and time required was strongest four days earlier (r=0.67, p<0.05). Additionally, a time lag with a decrease in the number of errors followed by a decrease in the time required was noted.

Comparing all pre- and post-intervention scores (Table 1), the FMA-UE was 51/66 points and 61/66 points, respectively. The scores showed significant improvement as follows (pre- to post-intervention): joint flexion (10/12 to 12/12), voluntary movement without joint movement (5/6 to 5/6), normal reflex (0/2 to 0/2), wrist joint (0/10 to 9/10), and coordination (4/6 to 5/6). For BBT and ARAT, improvements from 20 to 23 blocks and from 50/57 to 52/57 points were noted, respectively. Grip strength also showed significant improvement for the right hand from 16.3 kg to 20.1 kg and the left hand from 8.4 kg to 14.8 kg. However, SIAS showed no improvement from pre-intervention, with two points each for upper and lower extremity superficial sensation, as well as upper and lower extremity deep sensation (total: 8/12 points). Similarly, no improvements were noted for MAL and AOQ, with maintaining scores of 3.0 and 3.2, respectively.

**Table 1:** Comparison pre- and post-intervention. FMA: Fugl-Meyer Assessment Upper Extremity, BBT: Box and Block Test, ARAT: Action Research Arm Test, SIAS: Stroke Impairment Assessment Set, MAL: Motor Activity Log, AOU: Amount of Use, QOM: Quality of Movement

		Pre X+15	Post X+15
FMA-UE (point)	Reflex activity	4/4	4/4
	Flexor synergy	10/12	12/12
	Extensor synergy	6/6	6/6
	Volitional movement mixing synergies	6/6	6/6
	Volitional movement with little or no synergy	5/6	5/6
	Normal reflex activity	0/2	0/2
	Wrist	2/10	9/10
	Hand	14/14	14/14
	Coordination /Speed	4/6	5/6
BBT (blocks)		20	23
ARAT (point)		50/57	52/57
SIAS sensory function (point)	Light touch sensation in the UE	2/3	2/3
	Position sensation in the UE	2/3	2/3
	Light touch sensation in the LE	2/3	2/3
	Position sensation in the LE	2/3	2/3
grip force (R/L) (kg)		16.3/8.4	20.1/14.8
MAL (point)	AOU	3.0/5.0	3.0/5.0
	QOM	3.2/5.0	3.2/5.0

Upon reflection during the intervention task, the patient initially showed a lack of awareness of one's own body but developed a sense of touch in period B and a sense of kinesthesia in period A2. Specifically, the patient responded that “he made fewer mistakes” in period A1, “knew how his fingers felt” in period B, and “knew how his movements felt, and can do the task without looking” in period A2. Post-intervention, the slope of the index for a sense of agency increased from 1.1 to 1.2, indicating a slight increase in the quantitative index. Excessive grip force observed pre-intervention was no longer observed, and variability during object manipulation as well as frequency of falling objects decreased.

## Discussion

In this case study, errors due to excessive grip force (e.g., dropping objects) and large force fluctuations during object manipulation were observed. Therefore, rehabilitation for sensorimotor dysfunction of the paralyzed hand and fingers was performed using a wearable device that provides compensatory and synchronous feedback of tactile information from the hand through vibration stimulation. As a result, the FMA-UE, a comprehensive assessment of upper extremity function, increased by 10 points, indicating functional improvement. Given the nature of the paper, the minimal clinically important difference (MCID) and minimal detectable change (MDC) were used as indicators of clinically significant change. The MCID and MDC for FMA-UE were 9-10 points [34] and 5.2 points [35], respectively. In this case, the improved post-intervention results fulfilled the MCID and MDC criteria, thereby implicating these changes as clinically significant. Although MAL values are usually found to contribute to the increase in FMA-UE values [36], no change in MAL was observed in the present case. As such, we considered the effect of the intervention as the direct cause for the improvements in FMA-UE.

The use of a wearable device that detects vibration information generated when touching an object and provides real-time feedback information may be complementary to sensory information in cases with post-stroke sensorimotor deficits [25]. We intervened with this device to provide real-time somatosensory feedback to the patient by restoring contact information in the paralyzed finger with a vibrator and transmitting this information from the clavicle. Kitai et al. [37] reported that the FMA-UE of the paralyzed upper extremity improved from 25/66 to 36/66 points and that the sense of motion was improved when the same device was used on a stroke patient. Meanwhile, no changes in passive and active sensory function were observed in the present case. Previous studies have also included electroencephalographic measurements, showing that the device can be used to activate bilateral visual association areas, primary somatosensory areas, superior parietal lobes, and inferior parietal lobes (angular gyrus and supramarginal gyrus) [37]. Of these, the activation of the inferior parietal lobule is significant, since it is responsible for multisensory integration and the generation of a sense of agency. The involvement of the right supramarginal gyrus in the generation of a sense of agency has been demonstrated in a previous study [38]. Although this case study did not directly examine brain activity, it confirmed that the sense of agency of the paralyzed hand was improved after rehabilitation with this device.

In stroke patients, motor paralysis prevents the regression of one's sense of agency, resulting in prediction errors. A decreased sense of agency has been reported in patients with post-stroke motor paralysis [39]. Furthermore, an association between the severity of motor paralysis and a decreased sense of agency has been shown [40], whereas the sense of agency increases with recovery of motor paralysis [41]. In the present case, the patient's sense of agency was assessed from his introspective reports in the early stages of the intervention. During the task, the patient initially had no awareness of his own body or movement, but with the intervention of the device, he gradually became aware of the sensation in his fingertips, and eventually, his sense of movement was confirmed by his introspective report. Other studies have similarly shown that changes in the sense of agency were examined using an instructed task [30,42]. The results of the present study revealed that the slope of the sense of agency index increased, thus showing it was becoming more sharply defined. Miyawaki et al. showed that when the information content of sensorimotor cues is insufficient during motor control, cognitive cues are used in a complementary manner [43]. In other words, even when it is difficult to improve sensory function, it is possible to generate a sense of agency by supplementing it with cognitive cues. The 9-HPT was used for this task, in which the goal was to remove all nine pegs and place them on a plate. This goal was used as a cognitive cue [44], suggesting that even without sensory function improvement, setting and achieving the goal may have contributed to an increased sense of agency.

On the other hand, a sense of agency is known to play the role of mediator between sensory input and motor output [45]. Even without improvement in the passive sensation of the paralyzed hand, a sense of agency occurs when the sensory prediction associated with the motor intention and the actual sensory feedback are synchronized in time [46]. In the present case, an oscillator was placed on the clavicle, and sensory input was temporally synchronized with object grasping, resulting in the patient’s ability to detect object features with the affected hand. When performing the task, the temporal synchronization of visual information from the paralyzed hand and somatosensory information from supraclavicular vibration created a sense of agency that may have influenced motor function improvement. It is interesting to note that changes were also observed in the number of errors and time required when performing the 9-HPT. Moreover, the slope of the linear functional equation obtained from the means of the A1-B-A2 periods and the cross-lagged correlation analysis showed decreased values for the time required as the number of errors decreased. In repeated motor trials, sensory predictions are corrected on each trial, which is an important component of motor learning, as the brain acquires a mechanism that minimizes prediction error through this process [47]. Interestingly, the lack of grip force adaptation to the weight of an object is more likely due to unclear internal representations of the object’s physical properties rather than sensory deficits based on a previous study [48]. The task of grasping a pin and manipulating it to a plate is considered a dynamic task, in which predictive control of the grasping force becomes essential [49-51]. Predictive control of continuously changing forces during object manipulation can be regulated by anticipatory adaptation based on an internal model of body dynamics [52,53]. Similarly, perceptual generation by oscillators is involved in anticipatory coordination during object manipulation, and real-time somatosensory feedback can constantly update the internal model responsible for motor learning. This mechanism contributes to the accuracy of object manipulation, which may have led to a direct practice effect: a decrease in the number of errors and a subsequent reduction in the time required to complete the task.

Despite the findings of this case study, several limitations were noted. First, it was not possible to record whether the error occurred during pin grasping or peg manipulation, making it unclear as to which movement was improved. Second, since there was no association between sensory deficits and improvement in on-task errors in the present case, it is unclear whether errors might have occurred during a dynamic grasping task independent of sensory deficits (e.g., during peg manipulation and placement on the plate). Furthermore, we did not measure object grip force to determine whether there was excessive force during such a task. It is therefore necessary to investigate the adjustments for the grip force needed based on the object's weight. Lastly, changes in the sense of agency during the X+15 period were detected only by subjective introspection reports via reflection, whereas quantitative evaluation was performed after motor control had improved to some extent based on the difficulty of the task. Therefore, it is necessary to re-examine the quantitative evaluation procedure for the sense of agency. By resolving these issues, it will be possible to clarify whether the generation of contact timing perception using vibration information improves the sense of agency in stroke hemiplegic patients, its influence in the generation of predictive mechanisms in object manipulation, and its contribution to the improvement of upper limb dysfunction. Please note that this case study represents the characteristics of a specific patient and is thus difficult to generalize to other cases. Whether vibration-based generation of contact timing perception will contribute to clinical practice in other cases of hand sensorimotor deficits remains to be investigated in further studies in the future.

## Conclusions

In the current study, we used a wearable rehabilitation device that provides compensatory and synchronous tactile feedback via vibration to the hand of a patient with post-stroke sensorimotor dysfunction, resulting in stagnation of motor skills in the hand and fingers. Following the intervention, there was a notable improvement in the patient’s upper extremity motor function score, which had previously plateaued. In addition, there was a marked reduction in both excessive grip force during object grasping and the frequency of dropped objects. We believe that this rehabilitative approach using transducer-based perception generation could be an innovative tool to address sensorimotor deficits in the hand and fingers following stroke.
